# Indoor Companion Animal Poisoning by Plants in Europe

**DOI:** 10.3389/fvets.2020.00487

**Published:** 2020-08-07

**Authors:** Alessia Bertero, Paola Fossati, Francesca Caloni

**Affiliations:** ^1^Department of Environmental Science and Policy (ESP), Università degli Studi di Milano, Milan, Italy; ^2^Department of Health, Animal Science and Food Safety “Carlo Cantoni” (VESPA), Università degli Studi di Milano, Milan, Italy

**Keywords:** companion animals, indoor, plants, poisoning, toxicoepidemiology

## Abstract

Indoor plant poisoning poses serious threats to companion animals. One of the major reasons of this kind of hazard can be identified in the increased amount of time that the pets spend indoor, sharing the domestic environment with their owners. In this review, the toxic houseplants most commonly associated with companion animal poisoning in Europe and well-documented in the literature are emphasized. An analysis of the major and emerging plant species accountable for companion animal poisoning is proposed, in order to provide a framework of the factors influencing these incidents. Indeed, knowing the way substances may induce toxic effects in companion animals can be useful in allowing easier diagnosis and treatment processes. In conclusion, the Authors argue that a better characterization of the phenomenon, as well as of its extent, would be allowed by the availability of a centralized system for the data collection. Furthermore, better information and awareness on the issue may help developing a focused corrective approach to prevent indoor pet poisoning in Europe.

## Introduction

Poisoning incidents in companion animals related to indoor plant exposures are far from uncommon in Europe (1–5) (Table 1). Data from the European literature show that 5–6% (2, 3) to 11% (9) of the enquiries on pet poisoning received by poison centers are related to plants. These episodes involve both mature and younger animals which may be led to the ingestion of plant parts by boredom, behavioral alterations, or curiosity (5), and are becoming increasingly frequent nowadays since pets are more often allowed and kept indoor, sharing the domestic environment with their owner. This new habit has strongly influenced the type of poisonings registered in companion animals, leading to the emergence of new trends related to the increased risk of exposure to indoor poisoning agents (24).

**Table 1:** Toxic plants responsible for indoor poisoning of companion animals in Europe.

**Plant name**	**Class**	**Toxins**	**Animal**	**Countries**	**References**
*Anthurium* spp.	Oxalate-containing plants	Insoluble calcium oxalates	Companion animals	France, Italy	(1, 3, 6)
*Aucuba japonica*	Glycoside-containing plants	Iridoid glycoside aucubin	Dog	Italy	(3)
*Cycas revoluta*	Glycoside-containing plants	Azoglycosides (cycasin, macrozamin, and neocycasin); beta-N-methylamino-L-alanine; unidentified high-molecular- weight compound	Dogs	Italy, Sweden	(3, 7, 8)
*Cyclamen* spp.	Saponin-containing plants	Terpenoid saponins (saxifragifolin B, cyclamin)	Dog	Italy	(3)
*Dieffenbachia* spp.	Oxalate-containing plants	Insoluble calcium oxalates and trypsin-like protease	Companion animals	France, Italy, UK	(1, 9–11)
*Dracaena marginata*	Saponin-containing plants	Steroidal saponins and glycosides	Dogs and cats	France, Italy, Switzerland	(1, 3, 12)
*Euphorbia pulcherrima*	Diterpenoid-containing plants	Diterpenoid euphorbol esters and steroids	Companion animals	France, Germany, Italy, Switzerland, UK	(1, 3, 7, 13–17)
*Ficus benjamina*	Protease-containing plants	Ficin, furocoumarins, and ficusin	Dogs and cats	France, Italy	(1–3, 9)
*Lilium* spp.	Glycoalkaloid-containing plants	Steroidal glycoalkaloids (SGA) and steroidal saponins	Cats	France, Hungary, Italy, Switzerland, UK	(1–3, 7, 12, 18–20)
*Nandina domestica*	Cyanogenic glycoside-containing plants	Cyanogenic glycosides; protoberberine and berberine alkaloids	Dog	Italy	(6)
*Rhododendron* spp.	Grayanotoxin-containing plants	Grayanotoxin glycosides	Cats, dogs, rabbits, tortoises	France, Germany, Italy, UK	(1–3, 10, 13, 21–23)
*Spathiphyllum* spp.	Oxalate-containing plants	Insoluble calcium oxalates	Cats, dogs, rabbit, iguana	France, Italy	(1–3)
*Zantedeschia aethiopica*	Oxalate-containing plants	Insoluble calcium oxalates and proteolytic enzymes	Dog	Italy	(3)

Many house plants grown in Europe contain substances that are able, under certain conditions (i.e., quantities and parts of the plant ingested, vegetative stage of the plant, etc.), to induce toxic effects in companion animals (5) but plant poisoning is likely to be underdiagnosed and to pass unnoticed because of the non-specific clinical signs, of the difficulties for the owner to spot the animal ingesting the plant and of the general unfamiliarity with toxic plants of both owners and veterinarians. In this context, a systematic collection and analysis of the most common and emerging plants accountable for companion animal poisoning would be helpful to comprehend the factors influencing this phenomenon in order to put in place appropriate preventive measures (i.e., implementing campaigns to inform pet owners on potentially toxic plants) but also to help veterinarians in diagnosis and treatment processes. It should be noticed that, due to the absence of an European centralized veterinary poison center, the literature on this topic is still scarce and data frequently rely on single and uncoordinated reports. This can be a limit for this work, because of the objective difficulties faced in finding data on the phenomenon, but also its strength since, due to the lack of information available, a review on the topic may be even more needed and beneficial.

So, in this paper we will present an overview on the major and emerging plant species accountable for companion animal poisoning episodes in Europe.

## *Anthurium* Spp.

*Anthurium*, also known as flamingo plant, is a tropical American plant belonging to the *Araceae* family characterized by waxy looking decorative dark green leaves and grown as houseplants. The inflorescence is a spadix made up of small flowers disposed into a spiral and beneath the spadix develops a spathe generally lance-shaped. The fruits are berries of various colors. All parts of *Anthurium* contain insoluble calcium oxalates (25) that can cause oral irritation and edema, sialorrhea, vomiting, and dysphagia, if ingested (5).

In France, in 2003 the Center National d'Informations Toxicologiques Vétérinaires of the College of Veterinary Medicine in Lyon (CNITV) received 72 enquiries on *Araceae*, of which around 15% were related to *Anthurium* (1). Cases of flamingo plant poisoning concerning cats were also reported by the Poison Control Center of Milan (CAV) (3, 6).

## Aucuba Japonica

*Aucuba japonica* (Cornaceae family), commonly called spotted laurel or Japanese laurel, is an evergreen ornamental shrub native to Japan, China, and Himalayas. It is characterized by glossy, thick green leaves with different degrees of spotted variegation and purple small flowers with white anthers, grouped in clusters. The fruits are little (around 1 cm of diameter) globose red drupes. This plant contains the iridoid glycoside aucubin which can exert irritant effects causing, in case of ingestion of stems and leaves by pets, mild diarrhea, and vomiting (26, 27). Besides its irritant actions, aucubin possesses many pharmacological activities: indeed many studies have pointed out that this compound may exhibit antioxidant, anti-inflammatory, anti-fibrotic, anti-cancer, hepatoprotective, neuroprotective, and osteoprotective actions (28).

A case of *Aucuba japonica* concerning a dog was reported by the Italian CAV (3).

## Cycas Revoluta

*Cycas revoluta*, also known as sago palm or king sago, is a common ornamental evergreen palm-like plant that is native to Asia (China and Japan). Before being grown for ornamental reasons, this plant was used, and still is, as a source of food starch in underdeveloped countries (29). Sago palm shows separate male (which produce ellipsoid pollen cones) and female plants: female plants are able to generate seeds (characterized by a bright red color) only if there is a male plant in proximity. Nevertheless, these plants rarely bloom when grown indoor (30). Various toxins are responsible for poisoning episodes observed in animals as well as in humans (31): the azoglycosides macrozamin, neocycasin, and cycasin, the latter of which is converted by β-glucosidase to methylazoxymethanol (a teratogenic hepatotoxic and carcinogenic compound), the neurotoxic amino-acid beta-N-methylamino-L-alanine and an unidentified compound characterized by a high molecular weight (32–34). These toxins can be isolated in all parts of the plant, even if the highest concentrations are detected in seeds (33) and the ingestion of just one or two of them may be potentially fatal to a dog (35). The onset of clinical signs, mainly gastrointestinal (diarrhea, sometimes with blood, or constipation) and neurological signs, generally occurs few hours after ingestion; liver damage has also been reported, with hepatic enzyme alterations, hypoproteinemia, hypoglycemia, and thrombocytopenia (33). Moreover, cycad poisoning is generally associated with a high mortality rate (around 30%) (33, 36) and dog seems the most susceptible species (3, 36, 37).

In Europe, poisoning episodes have been reported in Italy (3, 7) and Sweden. In 2008 the Swedish Poisons Information Center received 3 enquiries on *Cycas revoluta* poisonings, all concerning dogs. Two animals were reported to show the typical gastrointestinal symptoms whereas the third case involved a 9-year-old dog that after the ingestion of *Cycas* roots displayed gastrointestinal symptoms together with severe disseminated intravascular coagulation (DIC) and had to be euthanized (8).

## *Cyclamen* Spp.

*Cyclamen* (Primulaceae family) is a genus grouping more than 20 species of wild and cultivated flowering perennial plants. *Cyclamen persicum*, the most famous species, is a common indoor plant cultivated for its charming white to red solitary flowers that grown on stalks. After blooming, a capsular fruit develops. The leaves are long-stalked and kidney or round shaped. This genus is characterized by the presence of toxic terpenoid saponins, i.e., saxifragifolin B and cyclamin (38) which are present in all parts of the plants, but especially tubers and roots. Cyclamen stems have been used in the indigenous medicine for their sedative, anthelmintic, laxative, and abortive actions (39) and different studies have shown that extracts of *Cyclamen* stems exhibit a large variety of biological activities (i.e., cytotoxicity, antimicrobial, and spermicidal properties) (38, 40, 41). The ingestion of this plant is followed by the onset of sialorrhea and gastrointestinal symptoms and, if large quantities of tubers are consumed, heart rhythm abnormalities, seizures, and death can also occur (42). In Italy, a case concerning a dog poisoning episode has been reported by CAV (3).

## *Dieffenbachia* Spp.

*Dieffenbachia* (dumbcane, Araceae family) includes around 30 species of tropical evergreen shrub. Some of them are characterized by large, variegated leaves, sometimes with yellowish marks on them, and are commonly grown indoor. The flowers born on long spadix, and the fruits are red or orange berries. The ingestion of stems or leaves cause the rapid development of sialorrhea and dysphagia due to the presence of insoluble calcium oxalates and a trypsin-like protease (43) which determine lip, tongue, palate, pharynx, and esophagus inflammation. Moreover, the swelling of the mucous membranes can lead to obstruction and respiratory impairment which may be fatal in severe cases (37, 44). Vomiting, diarrhea, keratoconjunctivitis, corneal ulceration, and eyelids edema are also observed (5). Indeed *Dieffenbachia* spp. (and other plants containing calcium oxalates) are characterized by the presence of cells called idioblasts that contain raphides (spicules of sharp shaped calcium oxalate crystals) stored in a gelatinous matrix formed by free oxalis acid. The lysis of the idioblasts (which occurs for example when the plant is chewed by an animal) lead to the swelling of the gelatinous material and that generates forces that causes the raphides to shoot out of the cells (process that continues for a large amount of time, even in the esophagus and in the stomach of the animal) with a great amount of energy, thus inducing a significant mechanical damage to the animal mucosa. Moreover, the presence of proteolytic enzymes induces the releases of histamine and proinflammatory cytokines which contribute to determine the tissue irritation and damage (45). The irritant properties of the oxalates, but also the induction of histamine release, seem to be the two mechanisms involved in *Dieffenbachia* intoxication. All domestic animals can be affected, but cats are particularly sensitive to this phytotoxins (5).

In France, in 2003 the CNITV received 72 enquiries on *Araceae*, and ~30% of these were related to *Dieffenbachia* (1), whereas a survey on the activity of CNITV by Keck et al. indicated this plant as a frequent culprit of small animal poisoning (9). Cases have been also reported in Italy to the CAV (10, 13). Some enquiries regarding the ingestion of these plants by companion animals (mainly dogs) have also been received by the Veterinary Poisons Information Service (VPIS) of London which reported for these episodes just mild to moderate clinical signs (11).

## Dracaena Marginata

The red-marginated dracaena, also called Madagascar dragon tree or cornstalk plant, is an ornamental plant from Madagascar characterized by clusters of twisted stems at the top of which develop rosettes of long, leathery green leaves with narrow reddish edges. Flowers are small and white, and the fruits are spherical yellow or orange berries, but they both only occasionally appear on indoor kept plants. The ingestion of any part of this plant may cause hypersalivation, gastrointestinal signs (mainly vomiting), weakness, incoordination, and mydriasis (the latter symptom has been described only in cats) due to the presence of steroidal saponins and glycosides (46, 47). In particular, 9 steroidal saponins (six of which identified as three pairs of 22-hydroxy furostanol saponins and their 22-methoxy derivatives) including 2 new minor compounds have been isolated from *D. marginata* (48). The exact toxicity mechanism of these compounds has not been fully understood yet, but is thought to be related to the irritant effects exerted on the gastrointestinal mucosa (27).

In France, around 3% of the calls on plants (*n* = 858) received by the CNITV in 2003 concerned Dracaena (1). Dog poisoning episodes due to this plant were also recorded in Switzerland (12). In Italy, the CAV recorded 3 enquiries on *Dracaena* poisoning episodes involving a cat, a dog and a rabbit, the latter with a fatal outcome (3).

## Euphorbia Pulcherrima

*Euphorbia pulcherrima* (Euphorbiaceae family) also known as poinsettia, is an ornamental plant native to Mexico and Guatemala, frequently used as a Christmas decoration due to its distinctive cream, pink, striped, or red leafy bracts, depending on the variety. These leaflike bracts (commonly mistaken for petals) are arranged around a central cluster of small yellowish flowers. This plant has a milky latex which is a strong irritant (37). The agents responsible for the harmful detergent-like effects, diterpenoid euphorbol esters, and steroids, act on the mucous membranes, gastrointestinal tract, and even on the skin (32, 37, 49), where they cause local irritation inducing vesicular dermatitis, conjunctivitis, stomatitis, vomiting, and diarrhea (5).

Even if this plant is considered of low toxicity (14) and deaths resulting from its ingestion have never been reported in small animals (49), poinsettia has a high potential as an agent of plant poisoning in Europe, due to its diffusion and attractiveness (5). Calls due to the ingestion of poinsettia by cats and dogs are received every year (mainly during the winter season) by the Veterinary Poisons Information Service (VPIS), but generally just mild gastrointestinal effects are reported, even if a case of a Burmese kitten who ingested a large leaf and developed severe oral and esophageal irritation has been described (14). In Italy, *Euphorbia pulcherrima* is a very common indoor ornamental plant that has been frequently found responsible for pet poisoning (7, 13). Amorena et al. described poinsettia as the most involved household plant in poisoning episodes in dogs (15) and another Italian study indicated this plant as one of the most frequently responsible for cat poisonings (16). Moreover, the CAV (3) received several enquiries during a 12-year period on cases of *Euphorbia pulcherrima* poisoning in companion animals (4 dogs and 8 cats) with hypersalivation, vomiting, diarrhea, and swelling being the most often reported symptoms. In France, around 5% of the enquiries on plants (*n* = 858) received in 2003 by the CNITV were related to *Euphorbiaceae* (1). In Switzerland, one poisoning case involving a dog has been reported (17) and, according to the data collected by five German poison center from 2012 to 2014 (21), *Euphorbia* was among the top five plants responsible for domestic animal exposures (*n* = 54).

## Ficus Benjamina

*Ficus benjamina* (Moraceae family), also known as weeping fig or Indian rubber plant, is native to India and Australia. It is a very common evergreen houseplant characterized by a brownish trunk (sometimes braided for ornamental reasons) surrounded by dense foliage. The leaves are glossy and elliptic, and the stems have milky sap. Weeping fig usually does not bloom or fruit indoors. *Ficus benjamina* possesses renowned medicinal activities which have been exploited by indigenous civilizations to treat several disorders (50). Indeed the leaves, cortex, and fruits contain many bioactive substances (i.e., cinnamic and caffeic acids, naringenin, quercetin, and stigmasterol) (50) but toxic compounds, namely ficin (a proteolytic enzyme), furocoumarins, and ficusin (a psoralen) (51, 52) have also been identified. These toxins, which have shown to possess many biological properties (i.e., cytotoxicity, antiviral, and antibacterial activities, etc.) (53–55), are thought to be responsible for the gastrointestinal and dermal irritation which can be observed after the exposure to this plant (52).

In France (1), this species has been described as the most frequently implicated in plant poisoning episodes, accounting for 43 calls (5% of the enquiries concerning plants) received by the CNITV, while Keck et al. in their survey on the activity of the center, reported *Ficus* among the often ingested plants by small animals (9). In Italy, Giuliano Albo, and Nebbia indicated *Ficus* spp. as one of the major culprit of cat poisoning episodes, among other plants (16), and the Veterinary Toxicological Assistance Service (SATV) described *Ficus* spp. as one of the major causes of cat poisoning episodes (56). *Ficus benjamina* has also been reported as a cause of plant poisoning in dogs by the CAV (10). In particular, two Italian surveys by CAV recorded cases of dog poisoning due to this plant (2, 3).

## *Lilium* Spp.

Lilies are herbaceous perennial plants belonging to the *Lilium* genus, which include 80–100 species native to temperate areas of the boreal hemisphere. They are characterized by leafy stems, long leaves, and solitary or clustered flowers made of six petallike segments, which may form elongate tube-like or globular shapes with a large variety of colors. The fruits are three celled capsules. Tiger lily (*Lilium tigrinum*) and other *Lilium* species (i.e., *L. longiflorum, L. speciosum, L. auratum*, etc.) are popular ornamental plants characterized by tubular flowers with projecting stamens, that can be found in gardens as well as in houses as potted plants or floral decorations. These plants are known to be nephrotoxic to cats, leading to acute renal failure due to tubular necrosis within 12–72 h after ingestion, while the onset of the initial symptoms (generally anorexia, lethargy, gastrointestinal signs, sialorrhea) can be observed 1–6 h after the exposure (57). The mechanism behind the renal tubular damage has not been fully understood yet, nevertheless, the induced nephrotoxic syndrome comprehends two phases: firstly a polyuric renal failure (12–24 h after ingestion) then, as a consequence, a severe dehydration occurs which lead to the onset of anuric renal failure (45). Several steroidal glycoalkaloids (SGA) and steroidal saponins have been found responsible for lily toxicity, and both leaves and flowers are toxic, but especially the latter (58); indeed the ingestion of 1 or 2 leaves or 1 flower has been reported to cause toxicosis (59). The mortality rate range between 5 and 100%, depending on the treatment (60). Cats are the only species known to develop renal failure after the ingestion of *Lilium*, dogs are not sensitive to these plants and just some cases with gastrointestinal signs have been reported, only after the ingestion of large quantities (32, 61). In France, in 2003 the CNITV received 133 enquiries related to *Liliaceae*, of which little <10% were due to *Lilium* exposure (1) and Fourez (18) reported a case of acute tubular necrosis and acidosis in a 7-month old domestic cat due to lily poisoning. In Italy, episodes of cat poisoning by *L. tigrinum* were recorded by the CAV (2, 3), and one case was reported to have a fatal outcome (10). Moreover, the CAV annual report for 2016 indicated *Lilium* as one of the most frequently involved plants in cases of animal poisoning (7). A lethal case of lily intoxication was also reported in an 8-month-old domestic cat in Hungary (19), one in Switzerland (12) and several poisoning episodes were recorded in UK (20).

## Nandina Domestica

*Nandina domestica* (Berberidaceae family), also known as sacred bamboo or heavenly bamboo, is a perennial broadleaf evergreen shrub native to China, Japan, and India, which is grown for its beautiful foliage and its impressive fruit display. In spring, white, tiny flowers appear in terminal clusters and in fall spherical, two-seeded, little red berries develop, lasting until the following spring. The fruits are particularly reach in cyanogenic glycosides, even if these toxins can be found also in other plant parts (62). After ingestion, cyanogenic glycosides are transformed into hydrogen cyanide (HCN) causing vomiting, dyspnea, cherry-red mucous membranes, respiratory failure, and convulsions. Cyanide is a mitochondrial toxin that affects cellular respiration, inhibiting the cytochrome oxidase along with many other enzymes, perturbing vital intracellular processes (63). Other identified toxic compounds are protoberberine alkaloids whose toxic significance is unknown and the alkaloid berberine which possesses anticholinesterase activity (64, 65).

In Italy, one case of a dog poisoned by *Nandina domestica* after a single oral exposure has been reported to the CAV during a 2-year period (6).

## *Rhododendron* Spp.

*Rhodendron* spp. (rhododendrons and azaleas, Ericaceae family) is a vast genus that includes over 850 species of plants (generally shrubs but also small trees) that are native to the north temperate zones of southeast Asia. They may be deciduous or evergreen plants, with spirally arranged leaves, and the most common species are characterized by clusters of large, colored flowers. The fruit is a dry capsule. This genus comprehends species that have been used in traditional medicine for the treatment of many diseases (i.e., inflammation, gastro-intestinal, and respiratory disorders, etc.) but some, due to their well know toxicity, have also been historically used as poisons (66). Many of these shrubs, that are now grown as ornamentals, can be found responsible for intoxication episodes in pets, showing similar clinical effects in all the domestic species (37). The compounds involved in *Rhododendron* toxicity are the grayanotoxin glycosides, that are present in all parts of the plant, including nectar (35). Eighteen grayanotoxins have been isolated and identified from plants belonging to the Ericaceae family but the main toxin in *Rhododendron* spp. is grayanotoxin I, which is also known as rhodotoxin, acetylandromedol, or andromedotoxin (22). These compounds act on the sodium channels of the cellular membranes, affecting both smooth, and striated muscles along with other excitable cells, leading to gastrointestinal, neurologic, and cardiovascular dysfunction. The poisoning symptoms are due to the continuous activation of sodium ion channels (thus the cells are continuously maintained in a state of depolarization and excitation, preventing the inactivation) that causes an increased vagal tone (67) with vomiting, diarrhea or constipation, dyspnea, cardiac alterations (tachycardia or bradycardia, arrhythmia, hypotension, and collapse), paralysis, and convulsions (5, 35, 68). The perturbation of the sodium channel function, which lead to intense calcium influx into the cells, cause a positive inotropic effect similar to that exerted by digitalis at low dosages, but the altered cardiac conductivity is also responsible for dysrhythmias and heart block (69).

In France, around 2% of the enquiries on plants (*n* = 858) received by the CNITV in 2003 regarded the Ericaceae family (1). Episodes of intoxication by *Rhododendron* in dogs, cats, and rabbits have been reported in Italy to the CAV (2, 3, 10, 13) and the animals displayed vomiting and weakness (3). In Germany, according to the analyses performed on the enquiries received by five German poison centers from 2012 to 2014 (21), *Rhododendron* spp. were among the top five plants involved in pet exposures (*n* = 35). Enquiries concerning the exposure of dogs to *Rhododendron* have also been received by the Veterinary Poisons Information Service (VPIS) of London: all the animals were symptomatic, showing lethargy, lassitude, ataxia, yawning, and head rubbing. One dog also displayed difficulties in picking up food and gaseous gastric efflux (22). In UK, intoxications by *Rhododendron* spp. have also been reported in tortoises: two affected animals showed severe lethargy after 12–24 h of ingesting rhododendron flowers but in both cases the outcome was favorable (23).

## *Spathiphyllum* Spp.

*Spathiphyllum* spp. or peace lilies belong to the *Araceae* family and are native to tropical rainforests of America and southeastern Asia. These are evergreen perennial plants with large glossy leaves and flowers (spadix) surrounded by white spathes that may cause, if ingested, oral irritation/burning, drooling, dysphagia, and vomiting, due to the presence of insoluble calcium oxalates (70). If a huge quantity of plant material is consumed, renal failure may also occur (57) but since *Spathiphyllum* induces stomatitis, the amount ingested is generally limited, so no renal signs are observed (71).

In France, the CNITV received in 2003 72 enquiries on *Araceae*, of which around 31% (the large majority, together with *Dieffenbachia*) concerned *Spathiphyllum* (1). Episodes of intoxication by *Spathiphyllum* in dogs, one rabbit and one iguana were also reported to the CAV (Italy) (2, 3).

## Zantedeschia Aethiopica

*Zantedeschia aethiopica* or calla lily is a rhizomatous herbaceous perennial plant belonging to the Araceae family, which is native to Southern Africa. It is a very common cut flower and also a popular potted plant characterized by large arrow-shaped leaves and flowers consisting of a yellow spadix surrounded by a white spathe. The fruit are orange-yellow globular berries carried in enlarged clusters.

The stems and leaves of this plant contain insoluble calcium oxalates crystals (raphides) which, due to the sharp shape, possess strong irritant effects toward the mucous membranes of mouth, tongue, pharynx, and esophagus (5, 72). Moreover, proteolytic enzymes are liberated by the plant cells when damaged, causing the release of histamine, and other inflammatory mediators (32). Clinical signs generally appear within 2 h of the ingestion and include oral hyperemia and edema, hypersalivation, anorexia, depression. Gastrointestinal signs (vomiting, diarrhea, abdominal pain) may also be present, especially if large quantities have been ingested (73) and sometimes dermatitis can occur as a result of dermal exposure to the plant (5). All the species are susceptible, but the cat is particularly sensitive (5).

In Italy a case of dog poisoning by calla lily was reported to the CAV (3).

## Treatment Strategy

Toxicity of the plants, besides being of course dependent on the species involved, is also influenced by many plant and environment related factors (5) such as the parts ingested, the vegetative stage, the environmental condition in which the plant is kept (i.e., watering, type of soil, temperature), etc. Indeed, these are all parameters that have a significant impact on toxin production and this aspect has to be considered when approaching a patient in order to correctly assess the severity of the intoxication and to set up a proper therapeutic strategy. In general, there are no specific therapies for plant intoxications, but knowing the species involved is very useful to emit a prognosis and to define how rapid has to be the medical intervention with decontamination and symptomatic/supportive care. Moreover, knowing the species implicated is advantageous because this information gives indication about the target organ of the toxin, helping to put in place a rapid and focused intervention. For example, according to Slater et al. in case of lily intoxication in cats, 87% of the animals that received prompt treatment (decontamination and intravenous fluid administration for kidney protection) did not developed renal signs or had just mild transient signs without consequences (60). Thus, immediate and aggressive veterinary care is crucial and make the difference in determining the survival rate and the quality and the rapidity of the recovery.

## Conclusions

Plants are frequently reported to the European Poison Control Centers as agents of companion animal poisoning but, notwithstanding the extent of the problem, this phenomenon has not been fully characterized yet due to the lack of a centralized system (on an international or even national level, in most cases) for the data collection and analysis of pet poisoning episodes, but also because many cases go undetected due to the non-specific symptoms and to the frequent owners and veterinarians lack of knowledge on the taxonomic aspects and toxic substances of the most common ornamental plants grown inside homes. Because information and awareness on the issue are fragmentary and imperfect, a systematic analysis performed on an international level may provide a global insight on the topic, raising attention, and also helping to finalize the efforts toward the development of a focused corrective approach based on the identification and in-depth knowledge of the emerging and common houseplants responsible for indoor pet poisoning in Europe. Furthermore, it may provide meaningful input for the development of regulatory proposal aimed at improving the availability of animal poisons center services and establishing systems for the recording of poisoning data.

## Author Contributions

AB and FC gave substantial contributions to the conception and design of the work. AB, FC, and PF drafted the work and revised it critically for important intellectual content and gave final approval of the version to be published and agreed to be accountable for all aspects of the work. All authors contributed to the article and approved the submitted version.

## Conflict of Interest

The authors declare that the research was conducted in the absence of any commercial or financial relationships that could be construed as a potential conflict of interest.

## References

[1] Barbier N Bilan D'activité du Centre National d'Informations Toxicologiques Vétérinaires Pour l'année 2003. Lyon: Thèse de doctorat vètèrinaire (2005).

[2] Caloni F Cortinovis C Rivolta M Davanzo F. Animal poisoning in Italy: 10 years of epidemiological data from the poison control centre of Milan. Vet Rec. (2012) 170:415. 10.1136/vr.100210 22271801

[3] Caloni F Cortinovis C Rivolta M Alonge S Davanzo F. Plant poisoning in domestic animals: epidemiological data from an Italian survey (2000-2011). Vet Rec. (2013) 172:580. 10.1136/vr.101225 23716536

[4] Cortinovis C Caloni F. Epidemiology of intoxication of domestic animals by plants in Europe. Vet J. (2013) 197:163–8. 10.1016/j.tvjl.2013.03.007 23570777

[5] Anadón A Martínez-Larrañaga MR Ares I Martínez MA Chapter 62 - poisonous plants of the Europe. In: Gupta RC, editor. Veterinary Toxicology. 3rd ed London: Academic Press (2018). p. 891–909.

[6] Le Mura A Intossicazioni da piante nel cane e nel gatto: casistica del centro antiveleni di milano (Master's degree). Università degli Studi di Milano, Milan, Italy (2018).

[7] Caloni F Cortinovis C Rivolta M Davanzo F Natural toxins: poisoning of domestic animal in Italy – 2016 annual report. Toxicol Lett. (2017) 280:S199 10.1016/j.toxlet.2017.07.865

[8] Holmgren A Hultén P The ancient plant cycas revoluta caused disseminated intravascular coagulation in a dog. Clin. Toxicol. (2009) 47:480 Available online at: https://apps.webofknowledge.com/full_record.do?product=WOS&search_mode=GeneralSearch&qid=1&SID=E1VZVDjcMH7CTWfgmXL&page=1&doc=1

[9] Keck G Berny P Buronfosse F Pineau X Vermorel E Rebelle B. Veterinary toxicovigilance: objectives, means and organisation in France. Vet Res Commun. (2004) 28:75–82. 10.1023/B:VERC.0000045382.46405.f3 15372933

[10] Berny P Caloni F Croubels S Sachana M Vandenbroucke V Davanzo F. Animal poisoning in Europe. Part 2: companion animals. Vet J. (2010) 183:255–9. 10.1016/j.tvjl.2009.03.034 19553146

[11] Campbell A Chapman M (editors). Dieffenbachia. In: Handbook of Poisoning in Dogs and Cats. Oxford: Blackwell Science (2000). p. 126.

[12] Schediwy M Mevissen M Demuth D Kupper J Naegeli H. New causes of animal poisoning in Switzerland. Schweiz Arch Tierheilkd. (2015) 157:147–52. 10.17236/sat00011 26753326

[13] Caloni F Scarpa P Pompa G Davanzo F Epidemiologia degli avvelenamenti degli animali domestici in Italia anni 2000-2002. Casistica Centro Antiveleni Milano Arch Vet. (2004) 55:1–6. Available online at: https://air.unimi.it/handle/2434/69655?mode=full.6#.Xx1-8_gzbJ8

[14] Campbell A Chapman M (editors). Poinsettia/*Euphorbia pulcherrima*. In: Handbook of Poisoning in Dogs and Cats. Oxford: Blackwell Science (2000). p. 55–6.

[15] Amorena M Caloni F Mengozzi G. Epidemiology of intoxications in Italy. Vet Res Commun. (2004) 28 (Suppl. 1):89–95. 10.1023/B:VERC.0000045384.00046.9b 15372935

[16] Giuliano Albo A Nebbia C. Incidence of poisonings in domestic carnivores in Italy. Vet Res Commun. (2004) 28:83–8. 10.1023/B:VERC.0000045383.84386.77 15372934

[17] Curti R Kupper J Kupferschmidt H Naegeli H. A retrospective study of animal poisoning reports to the swiss toxicological information centre (1997-2006). Schweiz Arch Tierheilkd. (2009) 151:265–73. 10.1024/0036-7281.151.6.265 19496046

[18] Fourez M Acute tubular necrosis and acidosis following lily poisoning in a young female cat. Point Vét. (2014) 45 (343 Part 1):12–6. Available online at: https://www.lepointveterinaire.fr/publications/le-point-veterinaire/article-canin/n-343/necrose-tubulaire-aigue-et-acidose-a-la-suite-d-une-intoxication-au-lys-chez-une-jeune-chatte.html

[19] Balka G Hetyey C Jakab C Lily poisoning in a cat. Case Rep Magyar Állatorvosok Lapja. (2011) 133:290–4. Available online at: https://apps.webofknowledge.com/full_record.do?product=WOS&search_mode=GeneralSearch&qid=8&SID=E1VZVDjcMH7CTWfgmXL&page=1&doc=2

[20] Sturgeon K Campbell A Lilium species poisoning in cats. Clin Toxicol. (2006) 44:521–2.

[21] McFarland SE Mischke RH Hopster-Iversen C von Krueger X Ammer H Potschka H. Systematic account of animal poisonings in Germany, 2012-2015. Vet Rec. (2017) 180:327. 10.1136/vr.103973 28235786

[22] Campbell A Chapman M (editors). *Rhododendron* and related plant species. In: Handbook of Poisoning in Dogs and Cats. Oxford: Blackwell Science (2000). p. 231–3.

[23] Brown S. Rhododendron species intoxication in *Testudo species* tortoises. Vet Rec. (2006) 158:742. 10.1136/vr.158.21.742 16731711

[24] Bertero A Fossati P Caloni F. Indoor poisoning of companion animals by chemicals. Sci Total Environ. (2020) 733:139366. 10.1016/j.scitotenv.2020.139366 32446086

[25] Guimaraes ALD do Tanque PR Pyrrho AD Vieira ACD Toxicological and anatomical study of vegetative organs of *Anthurium maricense* nadruz and mayo (*Araceae*). Rev Agrogeoambiental. (2019) 11:87–106. 10.18406/2316-1817v11n220191289

[26] Dey PM Harborne JB Plant Biochemistry. Amsterdam: Elsevier (1997).

[27] Knight A A Guide to Poisonous House and Garden Plants. New York, NY: Teton NewMedia (2007).

[28] Zeng X Guo F Ouyang D. A review of the pharmacology and toxicology of aucubin. Fitoterapia. (2020) 140:104443. 10.1016/j.fitote.2019.104443 31790767

[29] Lal JJ SAGO PALM. In: Caballero B, editor. Encyclopedia of Food Sciences and Nutrition. Oxford: Academic Press (2003). p. 5035–9.

[30] Missouri-Botanical-Garden (2020). Available online at: http://www.missouribotanicalgarden.org/PlantFinder/PlantFinderDetails.aspx?taxonid=279640 (accessed June 27, 2020).

[31] Chang SS Chan YL Wu ML Deng JF Chiu T Chen JC. Acute *Cycas* seed poisoning in Taiwan. J Toxicol Clin Toxicol. (2004) 42:49–54. 10.1081/CLT-120028744 15083936

[32] Botha CJ Penrith ML. Potential plant poisonings in dogs and cats in southern Africa. J S Afr Vet Assoc. (2009) 80:63–74. 10.4102/jsava.v80i2.173 19831265

[33] Clarke C Burney D. Cycad palm toxicosis in 14 dogs from Texas. J Am Anim Hosp Assoc. (2017) 53:159–66. 10.5326/JAAHA-MS-6517 28291394

[34] Forrester MB Layton GM Varney SM. *Cycas* revoluta (sago cycad) exposures reported to Texas poison centers. Am J Emerg Med. (2019) 38:P1611–5. 10.1016/j.ajem.2019.158446 31699425

[35] Milewski LM Khan SA An overview of potentially life-threatening poisonous plants in dogs and cats. J Vet Emerg Crit Care. (2006) 16:25–33. 10.1111/j.1476-4431.2005.00151.x

[36] Ferguson D Crowe M McLaughlin L Gaschen F. Survival and prognostic indicators for cycad intoxication in dogs. J Vet Intern Med. (2011) 25:831–7. 10.1111/j.1939-1676.2011.00755.x 21777288

[37] Cortinovis C Caloni F Plants toxic to farm and companion animals. In: Carlini CR Ligabue-Braun R, editors. Plant Toxins. Dordrecht: Springer Netherlands (2017). p. 107–34.

[38] El Hosry L Di Giorgio C Birer C Habib J Tueni M Bun SS. *In vitro* cytotoxic and anticlastogenic activities of saxifragifolin B and cyclamin isolated from *Cyclamen persicum* and *Cyclamen libanoticum*. Pharm Biol. (2014) 52:1134–40. 10.3109/13880209.2013.879600 24649909

[39] Speroni E Cervellati R Costa S Dall'Acqua S Guerra MC Panizzolo C. Analgesic and antiinflammatory activity of *Cyclamen repandum* S. et S. Phytother Res. (2007) 21:684–9. 10.1002/ptr.2145 17444577

[40] Caliş T Satana ME Yürüker A Kelican P Demirdamar R Alaçam R. Triterpene saponins from *Cyclamen mirabile* and their biological activities. J Nat Prod. (1997) 60:315–8. 10.1021/np960658j 9090874

[41] Mahasneh AM El-Oqlah AA. Antimicrobial activity of extracts of herbal plants used in the traditional medicine of Jordan. J Ethnopharmacol. (1999) 64:271–6. 10.1016/S0378-8741(98)00132-9 10363844

[42] ASPCA Cyclamen. (2020). Available online at: https://www.aspca.org/pet-care/animal-poison-control/toxic-and-non-toxic-plants/cyclamen (accessed May 24, 2020).

[43] Altin G Sanli A Erdogan BA Paksoy M Aydin S Altintoprak N. Severe destruction of the upper respiratory structures after brief exposure to a dieffenbachia plant. J Craniofac Surg. (2013) 24:e245–7. 10.1097/SCS.0b013e318286068b 23714978

[44] Loretti AP da Silva Ilha MR Ribeiro RE. Accidental fatal poisoning of a dog by *Dieffenbachia picta* (dumb cane). Vet Hum Toxicol. (2003) 45:233–9. 14513888

[45] Plumlee KH Chapter 25 – plants. In: Plumlee KH, editor. Clinical Veterinary Toxicology. Saint Louis, MO: Mosby (2004). p. 337–442.

[46] Rezgui A Mitaine-Offer AC Pertuit D Miyamoto T Tanaka C Delemasure S. Steroidal saponins from *Dracaena marginata*. Nat Prod Commun. (2013) 8:157–60. 10.1177/1934578X1300800205 23513716

[47] ASPCA Dracaena. (2020). Available online at: https://www.aspca.org/pet-care/animal-poison-control/toxic-and-non-toxic-plants/dracaena (accessed May 28, 2020).

[48] Ghaly N Nabil M Miyase T Melek F Steroidal saponins from the roots of *Dracaena marginata* tam. Der Pharmacia Lettre. (2014) 6:132–41. Available online at: https://www.scholarsresearchlibrary.com/abstract/steroidal-saponins-from-the-roots-of-dracaena-marginata-tam-9750.html

[49] Gwaltney-Brant SM Chapter 39 - christmastime plants. In: Peterson ME Talcott PA, editors. Small Animal Toxicology. 3rd ed Saint Louis, MO: WB Saunders (2013). p. 499–511.

[50] Imran M Rasool N Rizwan K Zubair M Riaz M Zia-Ul-Haq M. Chemical composition and biological studies of *Ficus benjamina*. Chem Cent J. (2014) 8:12. 10.1186/1752-153X-8-12 24524349 PMC4015825

[51] CABI Invasive Species Compendium - Ficus benjamina (Weeping Fig). (2014). Available online at: https://www.cabi.org/isc/datasheet/24065#A3D742BD-3A12-47C5-BE20-D6A1A2B2AB02 (accessed May 26, 2020).

[52] ASPCA Fig. (2020). Available online at: https://www.aspca.org/pet-care/animal-poison-control/toxic-and-non-toxic-plants/fig (accessed May 26, 2020).

[53] Almahy H Rahmani M Sukari MA Ali AM The chemical constituents of *Ficus benjamina linn*. and their biological activities. J Sci Technol. (2003) 11:73–81. Available online at: http://psasir.upm.edu.my/id/eprint/3830/

[54] Parveen M Ghalib RM Mehdi S Mattoo R Ali M A novel antimicrobial triterpenic acid from the leaves of *Ficus benjamina* (var. comosa). J Saudi Chem Soc. (2009) 13:287–90. 10.1016/j.jscs.2009.10.010

[55] Yarmolinsky L Huleihel M Zaccai M Ben-Shabat S. Potent antiviral flavone glycosides from *Ficus benjamina* leaves. Fitoterapia. (2012) 83:362–7. 10.1016/j.fitote.2011.11.014 22155188

[56] Giuliano Albo A Gusson F Scotti C Nebbia C Servizio di Assistenza Tossicologica Veterinaria: analisi retrospettiva delle chiamate registrate nel periodo 1996-2003. Il Progresso Vet. 3. Available online at: https://www.ordiniveterinaripiemonte.it/rivista/04n03/home.htm

[57] Panziera W Schwertz CI Henker LC Konradt G Bassuino DM Fett RR Lily poisoning in domestic cats. Acta Sci Vet. (2019) 47:357 10.22456/1679-9216.89516

[58] Uhlig S Hussain F Wisloff H. Bioassay-guided fractionation of extracts from easter lily (*Lilium longiflorum*) flowers reveals unprecedented structural variability of steroidal glycoalkaloids. Toxicon. (2014) 92:42–9. 10.1016/j.toxicon.2014.09.004 25269117

[59] Rumbeiha WK Francis JA Fitzgerald SD Nair MG Holan K Bugyei KA. A comprehensive study of easter lily poisoning in cats. J Vet Diagn Invest. (2004) 16:527–41. 10.1177/104063870401600607 15586568

[60] Slater MR Gwaltney-Brant S. Exposure circumstances and outcomes of 48 households with 57 cats exposed to toxic lily species. J Am Anim Hosp Assoc. (2011) 47:386–90. 10.5326/JAAHA-MS-5629 22058344

[61] Bates N Rawson-Harris P Edwards N. Common questions in veterinary toxicology. J Small Anim Pract. (2015) 56:298–306. 10.1111/jsap.12343 25728477

[62] Forrester MB. Pediatric nandina domestica ingestions reported to poison centers. Hum Exp Toxicol. (2017) 37:338–42. 10.1177/0960327117705429 28421827

[63] Bhattacharya R Flora SJS CHAPTER 19 - cyanide toxicity and its treatment. In: Gupta RC, editor. Handbook of Toxicology of Chemical Warfare Agents. San Diego, CA: Academic Press (2009). p. 255–70.

[64] Woldemeskel M Styer EL. Feeding behavior-related toxicity due to nandina domestica in cedar waxwings (*Bombycilla cedrorum*). Vet Med Int. (2010) 2010:818159. 10.4061/2010/818159 21197466 PMC3005831

[65] Colorado-State-University Guide to Poisonous Plants. (2019). Available online at: https://csuvth.colostate.edu/poisonous_plants/Plants/Details/117 (accessed May 22, 2020).

[66] Popescu R Kopp B. The genus *Rhododendron:* an ethnopharmacological and toxicological review. J Ethnopharmacol. (2013) 147:42–62. 10.1016/j.jep.2013.02.022 23454683

[67] Jansen SA Kleerekooper I Hofman ZLM Kappen I Stary-Weinzinger A van der Heyden MAG. Grayanotoxin poisoning: 'mad honey disease' and beyond. Cardiovasc Toxicol. (2012) 12:208–15. 10.1007/s12012-012-9162-2 22528814 PMC3404272

[68] Zoltani CK Chapter 14 - cardiovascular toxicity. In: Gupta RC, editor. Veterinary Toxicology. London: Academic Press (2018). p. 227–38.

[69] San Andrés Larrea MI San Andrés Larrea MD Rodriguez Fernández C. Plants, poisonous (animals). In: Wexler P, editor. Encyclopedia of Toxicology. 3rd ed. Oxford: Academic Press (2014) 960–9.

[70] ASPCA Peace Lily. (2020). Available online at: https://www.aspca.org/pet-care/animal-poison-control/toxic-and-non-toxic-plants/peace-lily (accessed June 5, 2020).

[71] Langston CE. Acute renal failure caused by lily ingestion in six cats. J Am Vet Med Assoc. (2002) 220:49–52, 36. 10.2460/javma.2002.220.49 12680447

[72] Kellerman TS Coetzer JAW Naudé TW Botha CJ Plant Poisonings and Mycotoxicoses of Livestock in Southern Africa. Cape Town: Oxford University Press (2005).

[73] Plumlee KH. Plant hazards. Vet Clin North Am Small Anim Pract. (2002) 32:383–95. 10.1016/S0195-5616(01)00006-7 12012742

